# Supplementary material for: P-TRAP: a Panicle Trait Phenotyping tool
Source: BMC Plant Biol. 2013 Aug 29;13:122. doi: 10.1186/1471-2229-13-122 (PMC3848748; doi:10.1186/1471-2229-13-122)
Supplement: Additional file 7 — P-TRAP source code. The Netbeans project that contains the source code of the application. [file 1471-2229-13-122-S7.zip › javadoc.zip!/javadoc/org-bioalg-riceprojecttype/org/bioalg/riceprojecttype/RiceProject.html]

RiceProject (RiceProjectType)


JavaScript is disabled on your browser.


- Package
- Class
- Tree
- Deprecated
- Index
- Help

*org.bioalg.riceprojecttype 1.0*

- Prev Class
- Next Class

- Frames
- No Frames

- All Classes

- Summary:
- Nested |
- Field |
- Constr |
- Method

- Detail:
- Field |
- Constr |
- Method


org.bioalg.riceprojecttype

## Class RiceProject

- java.lang.Object
- - org.bioalg.riceprojecttype.RiceProject

- All Implemented Interfaces:
  :   org.netbeans.api.project.Project, org.openide.util.Lookup.Provider

  ---

    

  ```
  public class RiceProject
  extends java.lang.Object
  implements org.netbeans.api.project.Project
  ```

  Author:
  :   faroq

- - ### Constructor Summary

    Constructors

    Constructor and Description

    `RiceProject(org.openide.filesystems.FileObject projectDir,
    org.netbeans.spi.project.ProjectState state)`
  - ### Method Summary

    Methods

    | Modifier and Type | Method and Description |
    | --- | --- |
    | `org.openide.util.Lookup` | `getLookup()` |
    | `org.openide.filesystems.FileObject` | `GetParticleResultFolder(boolean create)` |
    | `org.openide.filesystems.FileObject` | `getProjectDirectory()` |
    | `org.openide.filesystems.FileObject` | `getRiceFolder(boolean create)` |
    | `org.openide.filesystems.FileObject` | `getRiceGrainResultsMasterFile(boolean create)` |
    | `org.openide.filesystems.FileObject` | `getRiceImageFolder(boolean create)` |
    | `org.openide.filesystems.FileObject` | `getRiceResultsDetailsFile(boolean create)` |
    | `org.openide.filesystems.FileObject` | `getRiceResultsFolder(boolean create)` |
    | `org.openide.filesystems.FileObject` | `getRiceResultsImageFolder(boolean create)` |
    | `org.openide.filesystems.FileObject` | `getRiceResultsMasterFile(boolean create)` |

    - ### Methods inherited from class java.lang.Object

      `clone, equals, finalize, getClass, hashCode, notify, notifyAll, toString, wait, wait, wait`

- - ### Constructor Detail


    - #### RiceProject

      ```
      public RiceProject(org.openide.filesystems.FileObject projectDir,
                 org.netbeans.spi.project.ProjectState state)
      ```
  - ### Method Detail


    - #### getProjectDirectory

      ```
      public org.openide.filesystems.FileObject getProjectDirectory()
      ```

      **Specified by:**
      :   `getProjectDirectory` in interface `org.netbeans.api.project.Project`


    - #### getRiceFolder

      ```
      public org.openide.filesystems.FileObject getRiceFolder(boolean create)
      ```


    - #### getRiceImageFolder

      ```
      public org.openide.filesystems.FileObject getRiceImageFolder(boolean create)
      ```


    - #### getRiceResultsFolder

      ```
      public org.openide.filesystems.FileObject getRiceResultsFolder(boolean create)
      ```


    - #### getRiceResultsImageFolder

      ```
      public org.openide.filesystems.FileObject getRiceResultsImageFolder(boolean create)
      ```


    - #### GetParticleResultFolder

      ```
      public org.openide.filesystems.FileObject GetParticleResultFolder(boolean create)
      ```


    - #### getRiceResultsMasterFile

      ```
      public org.openide.filesystems.FileObject getRiceResultsMasterFile(boolean create)
      ```


    - #### getRiceGrainResultsMasterFile

      ```
      public org.openide.filesystems.FileObject getRiceGrainResultsMasterFile(boolean create)
      ```


    - #### getRiceResultsDetailsFile

      ```
      public org.openide.filesystems.FileObject getRiceResultsDetailsFile(boolean create)
      ```


    - #### getLookup

      ```
      public org.openide.util.Lookup getLookup()
      ```

      **Specified by:**
      :   `getLookup` in interface `org.netbeans.api.project.Project`

      **Specified by:**
      :   `getLookup` in interface `org.openide.util.Lookup.Provider`


- Package
- Class
- Tree
- Deprecated
- Index
- Help

*org.bioalg.riceprojecttype 1.0*

- Prev Class
- Next Class

- Frames
- No Frames

- All Classes

- Summary:
- Nested |
- Field |
- Constr |
- Method

- Detail:
- Field |
- Constr |
- Method
